# Predictors of incident malnutrition—a nutritionDay analysis in 11,923 nursing home residents

**DOI:** 10.1038/s41430-021-00964-9

**Published:** 2021-07-08

**Authors:** Gabriel Torbahn, Isabella Sulz, Franz Großhauser, Michael J. Hiesmayr, Eva Kiesswetter, Karin Schindler, Cornel C. Sieber, Marjolein Visser, Jasmin Weber, Dorothee Volkert

**Affiliations:** 1 Institute for Biomedicine of Aging, Friedrich-Alexander-Universität Erlangen-Nürnberg, Nuremberg, Germany; 2 Institute for Medical Statistics, Center for Medical Statistics, Informatics and Intelligent Systems, Medical University Vienna, Vienna, Austria; 3 Division Cardiac-, Thoracic-, Vascular Anaesthesia and Intensive Care, Medical University Vienna, Vienna, Austria; 4 Department of Internal Medicine III, Division of Endocrinology and Metabolism, Medical University Vienna, Vienna, Austria; 5 Kantonsspital Winterthur, Winterthur, Switzerland; 6 Department of Health Sciences, Faculty of Science, Amsterdam Public Health Research Institute, Vrije Universiteit Amsterdam, Amsterdam, The Netherlands

**Keywords:** Risk factors, Epidemiology

## Abstract

**Background/Objectives:**

Malnutrition (MN) in nursing home (NH) residents is associated with poor outcome. In order to identify those with a high risk of incident MN, the knowledge of predictors is crucial. Therefore, we investigated predictors of incident MN in older NH-residents.

**Subjects/Methods:**

NH-residents participating in the nutritionDay-project (nD) between 2007 and 2018, aged ≥65 years, with complete data on nutritional status at nD and after 6 months and without MN at nD. The association of 17 variables (general characteristics (*n* = 3), function (*n* = 4), nutrition (*n* = 1), diseases (*n* = 5) and medication (n = 4)) with incident MN (weight loss ≥ 10% between nD and follow-up (FU) or BMI (kg/m^2^) < 20 at FU) was analyzed in univariate generalized estimated equation (GEE) models. Significant (*p* < 0.1) variables were selected for multivariate GEE-analyses. Effect estimates are presented as odds ratios and their respective 99.5%-confidence intervals.

**Results:**

Of 11,923 non-malnourished residents, 10.5% developed MN at FU. No intake at lunch (OR 2.79 [1.56–4.98]), a quarter (2.15 [1.56–2.97]) or half of the meal eaten (1.72 [1.40–2.11]) (vs. three-quarter to complete intake), the lowest BMI-quartile (20.0–23.0) (1.86 [1.44–2.40]) (vs. highest (≥29.1)), being between the ages of 85 and 94 years (1.46 [1.05; 2.03]) (vs. the youngest age-group 65–74 years)), severe cognitive impairment (1.38 [1.04; 1.84]) (vs. none) and being immobile (1.28 [1.00–1.62]) (vs. mobile) predicted incident MN in the final model.

**Conclusion:**

10.5% of non-malnourished NH-residents develop MN within 6 months. Attention should be paid to high-risk groups, namely residents with poor meal intake, low BMI, severe cognitive impairment, immobility, and older age.

## Introduction

The number of nursing home (NH) residents was estimated by the number of beds at about 1.6 million in the US in 2015 and 4.0 million in Europe in 2017 [1]. NH-residents are at high risk of malnutrition which is reported in up to 54%, depending on the definition and cut-off value used [2–5]. Malnutrition is associated with poor health and functional outcomes as well as higher health care costs [6–11]. Therefore, malnutrition is a burden for the persons affected and for the health care system, and its prevention of utmost importance.

### Currently, little is known about the development of malnutrition in NH-residents over time

Many factors are associated with malnutrition in cross-sectional studies. In a systematic review, including studies with at least 100 NH-residents each, depression, poor oral intake, swallowing disorders, chewing problems, and eating dependency were associated with weight loss (WL) in 6 studies, and immobility and dependence in activities of daily living (ADL), poor oral intake, chewing problems, dysphagia and female sex were associated with low Body Mass Index (BMI) in 7 studies [12]. Since these cross-sectional results do not allow conclusions about the direction of the associations, longitudinal studies are needed. Up to now, only four longitudinal studies examined this association in NH-residents, which found cognitive decline [13], lower functional status/ higher dependency in ADL [14, 15], constipation [16], hospitalization [14], eating dependency [16], and low appetite [15, 16] as risk factors of malnutrition. These results are however based on rather small samples of 108 [15] to 441 [16] residents, and other eventually important risk factors, such as low food intake, might be missing. Most important, residents who were already malnourished at baseline were not excluded, resulting in different probabilities to develop malnutrition within each sample.

Based on repeated measurements data, a decline in nutritional status (based on low BMI, unintentional WL, low nutritional intake) was observed in 23% of NH-residents within 1 year [17]. A 2-year incidence of 26% according to the MNA-SF (<8 points) has been described [14], and for a period of 6 months, 26 and 2% incident malnourished cases according to weight loss (WL) of ≥2 kg [15] and >10% [16] have been reported.

Identification of factors increasing the risk and predicting the development of malnutrition is of utmost importance to help prevent malnutrition and its negative consequences. Thus, our aims were to examine the incidence of malnutrition and to identify predictors of incident malnutrition in a large sample of NH-residents.

## Materials/subjects and methods

### Reporting

This manuscript adheres to the reporting standards for observational studies (STROBE-statement (Supplemental material, Table 1)) [18, 19].

**Table 1:** Baseline Characteristics.

Variable	NH-residents with MN-data at FU (*n* = 11,923)
Age (years)	
65–74	1442 (12.1%)
75–84	3428 (28.8%)
85–94	5804 (48.7%)
95–107	1249 (10.5%)
Sex	
Male	3801 (31.9%)
Female	8122 (68.1%)
BMI (kg/m^2^)	
20.0–22.9	2981 (25.0%)
23.0–25.6	2981 (25.0%)
25.7–29.0	2993 (25.1%)
29.1–64.8	2968 (24.9%)
Mobility	
Mobile	4702 (39.4%)
Partially mobile	4274 (35.8%)
Immobile	2902 (24.3%)
Missing	45 (0.4%)
Cognitive impairment	
None	4334 (36.3%)
Slight to moderate	4793 (40.2%)
Severe	2761 (23.2%)
Missing	35 (0.3%)
Dysphagia	
No	10697 (89.7%)
Yes	1082 (9.1%)
Missing	144 (1.2%)
Chewing problems	
No	9742 (81.7%)
Yes	2032 (17.0%)
Missing	149 (1.2%)
Intake at lunch	
Three-quarters to all	8181 (68.6%)
Half	2623 (22.0%)
A quarter	624 (5.2%)
Nothing	143 (1.2%)
Nothing because of artificial nutrition	120 (1.0%)
Missing	232 (1.9%)
Cancer	
No	11380 (95.4%)
Yes	543 (4.6%)
Neurologic diseases	
No	3613 (30.3%)
Yes	8310 (69.7%)
Musculoskeletal diseases	
No	7808 (65.5%)
Yes	4115 (34.5%)
Cardiovascular/pneumological diseases	
No	5911 (49.6%)
Yes	6012 (50.4%)
Other diseases	
No	9421 (79.0%)
Yes	2502 (21.0%)
Antibiotics	
No	11433 (95.9%)
Yes	345 (2.9%)
Opiates	
No	10002 (83.9%)
Yes	1766 (14.8%)
Missing	155 (1.3%)
Psychoactive substances	
No	6322 (53.0%)
Yes	5460 (45.8%)
Missing	141 (1.2%)
Number of drugs	
<5 drugs	2714 (22.8%)
>=5 drugs	9043 (75.8%)
Missing	166 (1.4%)

NH nursing home, MN malnutrition, FU 6-months follow-up.

### Study design

This analysis is part of the Joint Action Malnutrition in the Elderly Knowledge Hub (MaNuEL) of the European Joint Programming Initiative A Healthy Diet for a Healthy Life (JPI‐HDHL) that investigated determinants and risk factors for (incident) malnutrition in older people in different settings [20–24] and is based on the nutritionDay-project (nD) (NCT04202939). The nD-project is a worldwide survey, performed annually in hospitals and NHs [25–27] with a follow-up (FU) after six months in the NH-setting [27]. The project is supported by the European Society for Clinical Nutrition and Metabolism (ESPEN) and national medical societies and is promoted via the internet, emails, newsletters, reports and at national and international congresses (e.g. of ESPEN and European Geriatric Medicine Society (EuGMS)). Resident and ward characteristics are assessed by standardized questionnaires which are available in more than 30 languages (https://www.nutritionday.org/). The nD-project was approved by the ethical committee of the Medical University of Vienna, Austria, by the ethical committee of the Friedrich-Alexander-Universität Erlangen-Nürnberg, Germany and by local ethical committees as required by national rules. All residents or their legal representatives gave written or oral consent prior to participation and all information was assessed completely anonymised.

### Inclusion and exclusion criteria

We included NH-residents aged 65 years or older participating in the nD-project between 2007 and 2018. Residents without data on body weight at FU were excluded as well as residents from Japan due to major differences in the nursing home system [28] and a consistently lower BMI in Japanese residents [29]. Further, residents with missing data on BMI or WL at baseline and those with malnutrition at baseline (defined by BMI <20 kg/m^2^ and/or WL of >5 kg in the past year (assessed via questionnaire)) were excluded to gain data on real incidence.

### Acquisition of data

Questionnaires were completed by local NH staff. Body weight in kilogram (kg) was measured using scales from the NH wards at nD and FU, or the most recent weight documented in the nursing records was used for both dates. NH staff was asked to measure body height using a stadiometer for residents who were able to stand. If this was not possible, height was extrapolated based on the measurement of knee height or was recorded from the information in nursing records or identification cards. BMI was calculated according to body weight (kg)/height (m)^2^. Height at nD was used for calculation of BMI at nD and FU.

### Outcome

Incident [30] malnutrition was defined as BMI < 20 kg/m^2^ and/or WL ≥ 10% between nD and FU, which was calculated as body weight difference between the two points in time.

### Potential predictors

Seventeen variables in 5 categories were included as potential predictors of incident malnutrition:

General characteristics (3 variables): age (65–74, 75–84, 85–94, 95–107 years), sex (female/male), BMI (quartiles: 20.0–22.9, 23.0–25.6, 25.7–29.0, 29.1–64.7 kg/m^2^).

Function (4 variables): mobility (mobile: able to leave the NH; partially mobile: able to get out of bed or chair without help of another person but unable to leave the NH unit; immobile: bound to a bed or (wheel)chair and unable to stand up without any help by other persons), cognitive impairment (none, slight to moderate, severe; classified by NH staff based on established classification criteria such as Mini Mental Status Test or, if not available, according to their subjective estimation), dysphagia (yes/no), chewing problems (yes/no).

Nutrition (1 variable): intake at lunch (three-quarters to all, half, a quarter, nothing, nothing because of artificial nutrition).

Diseases (5 variables): diagnosis of cancer (yes/no), neurologic diseases (e.g. dementia, stroke) (yes/no), musculoskeletal diseases (e.g. rheumatoid arthritis, osteoporosis) (yes/no), cardiovascular/pneumological diseases (yes/no), other diseases (yes/no).

Medication (4 variables): intake of antibiotics (yes/no), opiates (yes/no), psychoactive substances (yes/no) and number of drugs (<5/≥5).

### Statistical analysis

Potential predictors are described by median with minimum and maximum for continuous variables (age and BMI) and by absolute numbers, percentages for categorical variables.

For categorical variables with more than 0.1% missing values, we added a missing category. Cases with missing values in a variable with less than 0.1% missing values (tube feeding, parenteral nutrition, and cancer) and cases with missing values for age or sex were excluded.

To assess associations between potential predictors and incident malnutrition, we conducted multivariate generalized estimating equation (GEE) analyses with NH-ward as cluster variable. Each potential predictor was first analyzed for its association with incident malnutrition in univariate GEE analyses. All variables with a *p*-value *p* < 0.1 were tested for multicollinearity (variation influence factor (VIF)) and those with a VIF < 5 were added to the multivariate model. A *p*-value of *p* < 0.005 was defined as significant in the final model [31, 32]. We tested potential interactions according to sex. Results are presented as odds ratios (OR) with the corresponding 99.5%-confidence intervals (99.5%-CI). Complete case analyses were conducted as sensitivity analyses. To describe the accuracy of the final model, we present the area under the curve (AUC).

All statistical analyses were performed using R version 3.6.1.

## Results

**Fig. 1:**
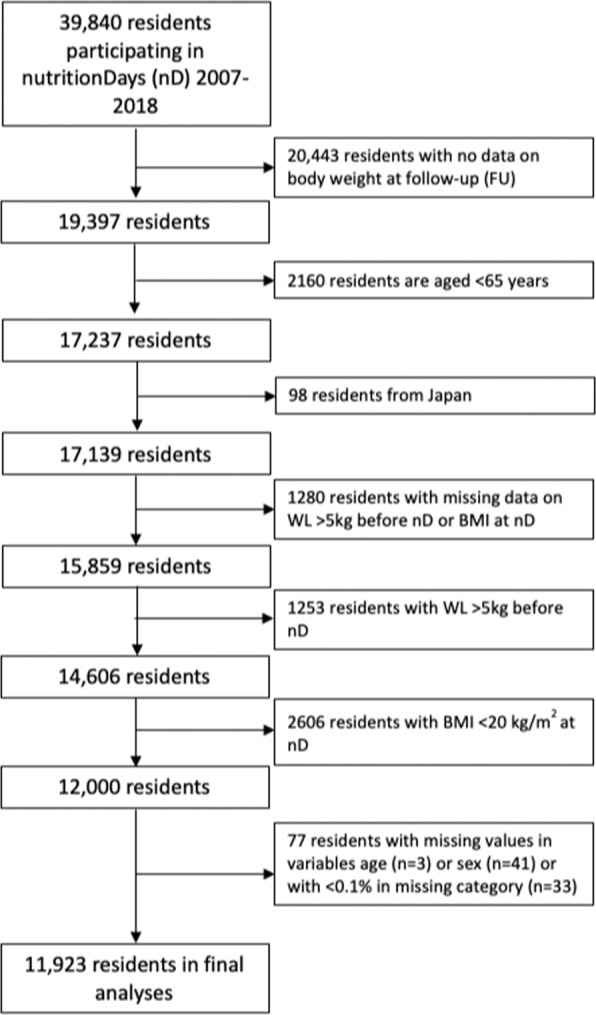
Study flow-chart. Flow of study participants.

Overall, 39,840 residents were assessed at nD between 2007 and 2018. For 20,443 residents, information on body weight at FU was missing, and 2,160 were <65 years of age. Ninety-eight residents from Japan were excluded as well as 1,280 residents with missing data on WL before nD or BMI at nD. 3,859 residents were malnourished at baseline (1,253 with WL > 5 kg before nD and 2,606 with BMI < 20 kg/m^2^ at nD) and thus also excluded. Seventy-seven with missing values in age or sex at baseline or because of a missing value in variables with < 0.1% missing values were excluded, resulting in 11,923 residents from North America and Europe included in the analyses (see flow chart, Fig. 1).

### Baseline characteristics

Median age was 86.0 [65.0–107.0] years and 68.1% were female. Median BMI was 25.7 [20.0–64.8] kg/m^2^, 24.3% were immobile and 23.2% had severe cognitive impairment. Further characteristics are described in Table 1.

### Incident malnutrition

At 6-month FU, 953 residents (8.0%) had a WL ≥ 10% and 586 (4.9%) had a BMI < 20 kg/m^2^, while in 290 residents (2.4%) both criteria were present simultaneously. Therefore, 1,249 (10.5%) residents developed malnutrition according to one or both of the two criteria.

### Predictors of incident malnutrition

**Fig. 2:**
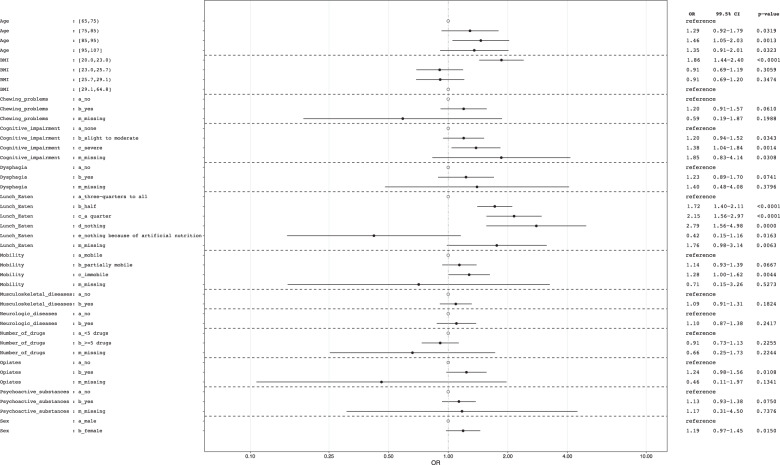
Chance for incidence of malnutrition according to different patient characteristics. Results of multivariate GEE-analyses (*n* = 11,923); OR: Odds ratio; 99.5%-CI: 99.5%-Confidence interval; cases with a percentage of < 0.1 in missing category and those with missing values in age and sex were excluded from analyses (*n* = 77).

All potential predictors except tube feeding, parenteral nutrition, cancer, cardiovascular/pneumological diseases, other diseases and antibiotics were associated with incident malnutrition in the univariate model with *p* < 0.1 (Supplemental material, Table 2). There was no indication for multicollinearity. Interaction according to sex acquired significance only for BMI-group 20.0–22.9. For male NH residents in this subgroup, the odds for incident malnutrition was higher than for females. The multivariate model included 13 variables univariately associated with incident malnutrition (AUC 0.68). No intake at lunch (OR 2.79 [1.56–4.98]), a quarter (OR 2.15 [1.56–2.97]) or half of the meal eaten at lunch (OR 1.72 [1.40–2.11]) (vs. three-quarter to complete intake), the lowest BMI-quartile (20.0–22.9 kg/m^2^) (OR 1.86 [1.44–2.40]) (vs. highest BMI-quartile (29.1–64.8 kg/m^2^)), the age-group 85–94 years (OR 1.46 [1.05; 2.03]) (vs. 65–74 years)), severe cognitive impairment (OR 1.38 [1.04; 1.84]) (vs. none) and being immobile (OR 1.28 [1.00–1.62]) (vs. mobile) predicted incident malnutrition in the final model (Fig. 2). In the sensitivity analysis including only complete cases (*n* = 11,170), associations and effect sizes were similar, only immobility could not retain significance (Supplemental material, Fig.1).

## Discussion

In a large, international sample of 11,923 non-malnourished NH-residents, 10.5% developed malnutrition within 6 months. Among 17 potential predictors, no or limited food intake at lunch, low BMI, severe cognitive impairment, immobility, and age 85–94 years were identified as predictors of incident malnutrition.

We defined malnutrition as low BMI and/or a significant WL, based on international agreement for several analyses within the MaNuEL Knowledge Hub. These variables represent two phenotypic criteria of the Global Leadership Initiative on Malnutrition (GLIM) global consensus definition [33], are widely used and were selected because of their availability in several data sets used for secondary data analyses. Complete information to apply the GLIM definition, e.g. muscle mass, is currently unfortunately not available in the nD data set.

The incidence of malnutrition at 6 months according to our definition was 10.5%, with 8.0% according to the WL-criterion and 4.9% according to the low BMI-criterion. Data on incident malnutrition in NH-residents is scarce and hardly comparable due to different definitions of malnutrition, different methods to assess malnutrition (e.g. measurement vs. self-report), and different FU-periods. Beck reported a WL of ≥10% within 6 months in only 2.1% of NH-residents [16] and Mamhidir et al. [34] reported a WL of ≥10% in 14% of older adults, however, within 1 year and in sheltered housing. In contrast to these studies, we excluded residents with malnutrition at baseline to consider only those with a potential risk to develop malnutrition and thus report true incidence. Although Beck [16] reported a markedly lower percentage of residents with a measured WL of ≥10% within 6 months, our data might be underestimated due to a high number of residents without FU-data, since residents with missing body weight and those who died, which were excluded from our sample, might have developed malnutrition more often. Nursing homes or wards that did not take part at the FU assessment might have been less interested in nutritional issues which could affect a poorer nutritional management when compared to nursing homes or wards with FU.

Residents aged 85–94 years were more likely to develop malnutrition compared to residents aged 65–74 years. While chances for the other age groups were also higher compared to the reference group, they did not show significance which could be due to the statistical power: nearly half of all participants were in the age group 85–94 years (see Table 1). Other prospective studies in NH did not find a significant association between age and (incident) WL [15] or deteriorating nutritional status according to MNA-SF [14]. In contrast to these analyses, age—and also BMI—were entered as categorical variables in our models, as results might be easier for interpretation and applicability in the clinical setting, and effects might be different for lower and higher values.

In our analyses, the lowest BMI-group (20.0–22.9) had a 1.86 times higher chance of incident malnutrition (Fig. 2) which seems reasonable, as residents with a BMI close to the cut-off of 20 kg/m^2^ at baseline may be more likely to develop malnutrition according to our definition than those with a higher BMI. Previous studies also reported this association, although they did not exclude residents with malnutrition at baseline [15, 35].

Dietary intake was assessed by observation during the lunch on nD. Overall, 22.0% consumed only half of the served portion, 5.2% a quarter, and 1.2% ate nothing at all. The chance of developing malnutrition increased, the lower the intake at lunch (Fig. 2). Unfortunately, intake at lunch is the only meal assessed at nD. Intake at lunch may depend on habits formed earlier [36], the timing of meal in the NH [37], and may differ from country to country due to different cultural habits [38]. However, Hiesmayr et al. showed that food intake assessed via plate diagrams was similar for breakfast, lunch, and dinner for patients in hospitals [25]. Additionally, in NH lunch is an important main meal and is often the only warm dish during the day [39] in many countries which suggests that this meal is an important indicator of daily food intake. Our results support those from cross-sectional studies showing that poor oral intake is associated with malnutrition, low BMI and WL [12, 40], emphasizing the importance of adequate food intake.

An adequate nutritional intake might not only lower the risk of malnutrition but also of mortality. In an earlier analysis from the nD-project, Streicher et al. showed that a low intake at lunch was associated with a higher risk of mortality in malnourished residents [41]. It has also been shown that mortality of NH-residents is predicted by WL and low BMI [42]. Therefore, future analyses should investigate whether WL and low BMI are mediators of the association between low food intake and mortality. Low food intake may be caused by a variety of factors such as functional limitations, psychological factors, or end-of-life situation. Thus, for a better understanding, also reasons for low or no intake need to be evaluated further.

Residents with severe cognitive impairment showed a 1.4-fold chance for incident malnutrition compared to those with a good cognitive status. The chance was also increased for those being slightly to moderately impaired but the result was not significant. Lannering et al. also showed that cognitive impairment at baseline is associated with malnutrition at FU [13]. It is well known and confirmed by recent studies, that a poor cognitive status is associated with low meal/energy intake [43, 44]. The occurrence of nutrition/mealtime-related problems such as olfactory and taste dysfunction, attention deficit, behavioral problems, or refusal to eat have repeatedly been described with declining cognitive abilities [45]. A longitudinal study reported a significant relationship between cognitive dysfunction and a higher risk for WL [13] but causal pathways should be further investigated.

Further, immobile residents had a higher chance (OR 1.28 [1.02–1.59]) to develop malnutrition compared to mobile residents. This is in line with results from a systematic review of cross-sectional studies which reported an association between low BMI and immobility in several studies [12]. Two longitudinal studies that investigated the association between baseline characteristics and malnutrition at FU also reported a significant association between poor functional status and malnutrition [14, 15]. In residents with dementia, problems with bringing food to the mouth are associated with loss of weight [46], while an uncomfortable position during the meal might deteriorate food intake in immobile residents [47]. In the sensitivity analysis, the association between immobility and incident malnutrition was similar but a little bit smaller (OR 1.25 [1.00–1.57]), and significance was not retained. One explanation might be the loss of power due to the exclusion of *n* = 753 residents with missing values in any of the included variables. For all other variables, results have not been changed, showing the robustness of our results in general.

Depending on the malnutrition screening tool used in the NH-setting [48], our identified predictors for incident malnutrition are already assessed during malnutrition screening but some tools do not consider any of these risk factors. Therefore, future studies should further investigate whether a low intake at lunch, a low BMI, immobility, severe cognitive impairment, and older age might further improve malnutrition screening tools in NH-residents.

### Strengths and limitations

Our study has several strengths. First, we could include a very large sample from different countries and different care systems. The questionnaires were standardized and available in different languages. Second, we present data on incident malnutrition which are very rare so far by excluding residents with malnutrition at baseline to reduce the risk of wrong causation. Third, we were able to consider a large set of variables from different domains to investigate the potential prediction of incident malnutrition. Moreover, we report our analyses according to a standardized reporting guideline (STROBE-statement) to ensure transparent and thorough reporting.

However, when interpreting our results, several limitations must be considered. First, data were assessed by local staff and not by trained scientific personnel. Data presented here are mostly assessed as part of daily routine with simple and quick methods and not via validated scales (such as Katz or Barthel Index for ADL). Our analyses are limited to the variables available in the nD dataset, and eventually relevant potential predictors, e.g. ADL, multimorbidity, or hospitalisation, could not be taken into account. Food intake on nD was assessed estimating the consumed amount of food via a plate diagram and not via dietary records or other more valid—but also more time-consuming—dietary assessment methods [49]. Thirdly, follow-up data at 6 months was not available for many residents, and we cannot exclude a respective selection bias. In addition, external validity may be limited due to selection bias by exclusion of residents with missing data on WL or BMI at baseline. Also, by inclusion of only those NH with FU, which might have been more interested in nutritional issues, the incidence of malnutrition might have been underestimated and may limit external validity. Finally, all observational studies are open to residual and unmeasured confounding [50].

## Conclusions and implications

In this large nD data set, 6-month incidence of malnutrition in nursing-home residents was 10.5%. A low intake at lunch, a low BMI, immobility, severe cognitive impairment and older age were identified as predictors for the development of malnutrition.

### Implications for practice

While older age represents a non-modifiable factor, it is also of importance since special attention should be given to older residents to timely start with respective preventive interventions. In contrast, functional and cognitive impairments represent at least partly modifiable risk factors. Beneficial effects of physical exercise and cognitive training interventions are on physical and cognitive status are well known, which might also affect the nutritional status positively. In case of severe functional or cognitive impairment, at least special attention should be given to these residents. Food intake also represents a modifiable risk factor, and if food intake (at lunch) is low, effective nutritional care strategies with a potential to prevent malnutrition (e.g. feeding assistance, pleasant environment, or sharing of mealtimes with others) are available and should be offered.

### Implications for research

Further prospective, large-scale cohort studies in nursing homes with longer follow-up periods and repeated measurements of nutritional status including reasons for WL (e.g. voluntary vs involuntary) and reasons for low intake should be conducted to complement and extent our results. In addition, food intake should be assessed more detailed, and reasons for drop-out should be recorded. Finally, it should be investigated whether inclusion of our identified risk factors in malnutrition screening tools will improve nutritional care and health outcomes.

## Supplementary information

Supplemental material
